# Genetic Insights Into Retinitis Pigmentosa: A De Novo *RPGR* Mutation in an Iranian–Azeri Family

**DOI:** 10.1155/crop/7640514

**Published:** 2026-08-17

**Authors:** Narges Ghezelbash, Mortaza Bonyadi, Mohammad Hossein Jabbarpour Bonyadi

**Affiliations:** ^1^ Animal Biology Department, Faculty of Natural Sciences, University of Tabriz, Tabriz, Iran, tabrizu.ac.ir; ^2^ Center of Excellence for Biodiversity, Faculty of Natural Sciences, University of Tabriz, Tabriz, Iran, tabrizu.ac.ir; ^3^ Ocular Tissue Engineering Center, Ophthalmic Research Center, Shahid Beheshti University of Medical Sciences, Tehran, Iran, sbmu.ac.ir

**Keywords:** RCC1-like domain, retinitis pigmentosa, *RPGR* gene, whole exome sequencing

## Abstract

Retinitis pigmentosa (RP) is a genetically heterogeneous disease characterized by progressive vision loss and blindness, primarily due to the degeneration of cone photoreceptor cells. The classic symptoms include a decline in visual acuity and retinal atrophy.

In this study, whole‐exome sequencing (WES) was performed on a 40‐year‐old man from a nonconsanguineous Iranian–Azeri Turkish family, who was diagnosed with vision loss and blindness at age 40. DNA was extracted from the proband, his healthy brother, sister, and mother to conduct segregation analysis. WES analysis revealed a hemizygous mutation in a single nucleotide (NC_000023.11)(NM_001034853.2) c.194G>T, located in exon 3 of the *RPGR* gene. In silico analysis suggested that this mutation likely activates a new cryptic donor site. Segregation analysis confirmed that the variant was absent in the mother and healthy siblings, indicating that it is a de novo mutation. We also screened a cohort of 430 healthy individuals from the same ethnic group, finding no occurrence of the variant. The presence of this de novo mutation, its absence among controls from the same ethnic background, and bioinformatics analysis collectively suggest the pathogenicity of the identified variant.

## 1. Introduction

Retinitis pigmentosa (RP) is a retinal neurodegenerative disorder characterized by the progressive loss of vision due to the impairment of rod photoreceptors, loss of cone photoreceptors, and atrophy of the retinal pigment epithelium (RPE) [1–3]. The most common symptoms include decreased central vision, tunnel vision, and night blindness (nyctalopia) [2, 4, 5]. The prevalence of RP is reported to be approximately 1 in 4000 worldwide, affecting more than 1.5 million individuals [1, 6, 7].

Over 80 different genes, including *RHO*, *RP2*, *USH2A*, and *RPGR*, have been implicated in the development of RP, highlighting its genetic heterogeneity [8–10]. The disease exhibits various inheritance patterns among affected families, including autosomal dominant (30%–40% of RP patients), autosomal recessive (50%–60% of RP patients), X‐linked (5%–15% of RP patients), and mitochondrial forms [11, 12]. Generally, X‐linked RP tends to present a more severe phenotype compared to autosomal recessive RP, whereas patients with autosomal dominant RP often have a better long‐term prognosis regarding the retention of central vision [13, 14].

In this report, we describe a de novo variant characterized as NC_000023.11 (NM_001034853.2):c.194G>T (p.G65V) in the *RPGR* gene. This mutation possibly creates a new cryptic donor site in the third exon and is associated with severe clinical manifestations in a 40‐year‐old patient with advanced RP. The observation of this de novo mutation, coupled with the patient′s severe vision loss and bioinformatics analyses, collectively supports the pathogenicity of the variant.

## 2. Materials and Methods

**Figure 1:**
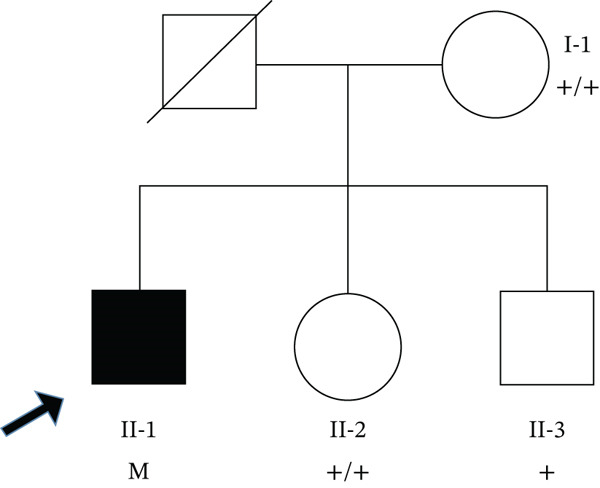
The pedigree of the patient. The proband is the offspring of a nonconsanguineous marriage. The healthy mother (I‐1) is genotyped for the variant as homozygous wild type (+/+), the proband (II‐1) is genotyped as hemizygous mutant (M), healthy siblings (II‐2, II‐3) are genotyped homozygous wild type (+/+) and hemizygous wild type (+), respectively.

A 40‐year‐old man diagnosed with RP was referred for genetic analysis. WES was employed to identify pathogenic genes, given the genetic heterogeneity of the disorder. The patient′s parents were nonconsanguineous, his siblings were unaffected, and his father had passed away (Figure 1). Blood samples were collected from the patient, the mother, and both healthy siblings (a brother and a sister), and genomic DNA was extracted for whole‐exome sequencing and subsequent Sanger validation [15, 16].

The WES analysis utilized the Agilent SureSelect Human All Exon V7 Target Enrichment kit. Sequencing of the enriched library was performed on an Illumina NovaSeq 6000 platform, generating paired‐end 150‐bp sequence reads. These reads were mapped to the UCSC human reference genome (GRCh37/hg19 and GRCh38/hg38 assemblies) using Burrows–Wheeler Aligner (BWA) and CLC Genomics Workbench [16].

Low‐quality reads (Qbase < 20) and duplicates were filtered out using Picard and Trimmomatic V0.39 software [15]. BAM files were sorted and indexed with Samtools. Single nucleotide variations (SNVs) and minor insertions or deletions (Indels) were identified in parallel using DeepVariant V1.1.0 and the Genome Analysis Toolkit (GATK v. 4.2.0.5). Variants were annotated using the Wannovar online tool [17].

An internal variant filtering process, based on ACMG and Sherloc standards, was applied to identify disease‐causing variants. Initially, variations were filtered based on minor allele frequency (MAF) from several population databases, including Iranome, gnomAD, 1000 Genomes, ExAC, and dbSNP. Common variants with MAF < 0.05 were retained. Although flanking exon areas were included, deep intronic, upstream/downstream, and synonymous variants were excluded. Functional implications, ClinVar findings, and multiple bioinformatics predictions were used to prioritize the remaining variants.

To evaluate structural and functional consequences, Human Splice Site Finder, MaxEntScan, NNSPLICE, and GeneSplicer were utilized. This analysis evaluated the impact of mutation on splice sites, branch points, and auxiliary sequences [18–20].

Sanger sequencing was performed on the proband and unaffected family members (mother, sister, and brother) using an ABI 3500 Genetic Analyzer (Applied Biosystems, Foster City, United States), following PCR amplification of genomic DNA with specifically designed primers targeting the variant region. The RNAsnp Web Server was used to assess the mutation′s effect on RNA secondary structure [21].

DynaMut was used to assess the identified mutation′s effect on RPGR protein stability and dynamics [22]. Structural differences between wild‐type and mutant forms were compared. Additionally, domain conservation across species was analyzed using UniProt [23].

## 3. Ethics Statement

This project was ethically approved by the University of Tabriz (IR.TABRIZU.REC.1402.161), and written informed consent was taken from all participants.

## 4. Results

**Figure 2:**
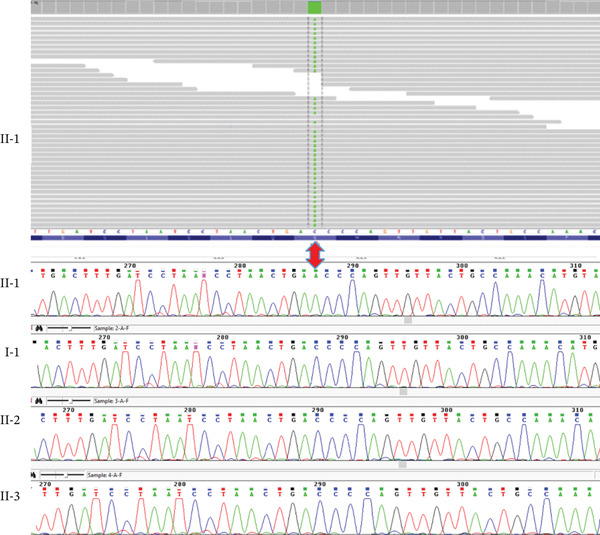
Whole exome sequencing results and Sanger sequencing results in the proband and his mother. The pathogenic variant in the *RPGR* gene (c.194G>T) is illustrated in the IGV figure of the BAM file of the proband (upper figure, II‐1). This potentially pathogenic variant is shown in a hemizygous state in the proband (upper graph, II‐1) and a healthy homozygous state in his mother (lower graph, I‐1) and healthy siblings: brother (II‐2) and sister (II‐3).

WES analysis revealed a hemizygous transition from G to T at position 194 (c.194G>T) (p.Gly65Val) within exon 3 of the *RPGR* gene located on chromosome X. This finding was further confirmed by Sanger sequencing (Figure 2A). Segregation analysis revealed that both siblings are homozygous for the wild‐type allele (c.194G>T), confirming they do not carry the pathogenic variant and are unaffected. The corresponding chromatograms for both siblings, along with those of the proband and the mother, are now presented in Figure 2B–D for direct comparison. The proband is married but has no children at the time of this report.

Using the Human Splicing Finder web tool, we observed that the mutation possibly created a new cryptic donor site within exon 3 of the RPGR gene, with a predicted activation probability of approximately 69%. This alteration may result in several significant molecular consequences, including altered protein function due to potential misfolding, splicing variations leading to dysfunctional isoforms, and an increased mutation rate that could contribute to retinal degenerative diseases. Furthermore, the emergence of this cryptic site may disrupt regulatory mechanisms that affect gene expression levels and interfere with critical protein interactions within retinal pathways. These findings underscore the importance of understanding the molecular implications of such genetic modifications in the context of retinal health and disease.

In silico modeling of the transcribed mRNA using the RTH web server indicated that the secondary structural features of the mutant mRNA transcript did not differ significantly from those of the wild‐type mRNA (data not shown). However, the minimum free energy of the mutant mRNA was −91.20 kcal/mol compared with −91.40 kcal/mol for the wild‐type mRNA. This notable alteration in minimum free energy suggests potential functional changes resulting from the genetic variant.

To examine the differences between the wild‐type and mutant proteins, additional computational analysis of protein structure was conducted using the DynaMut web server. The results demonstrated changes in the hydrogen bonding patterns between amino acid residues in the mutant protein compared to the wild‐type.

**Figure 3:**
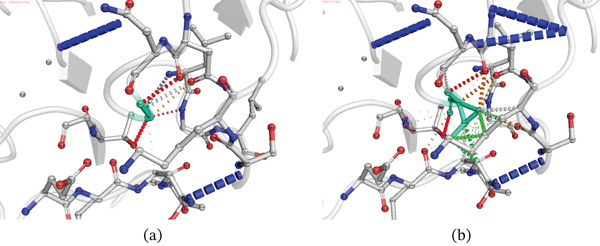
Comparative 3D structural analysis of the local interatomic interaction networks in wild‐type (WT) and mutant (MT) RPGR protein. (a) WT RPGR: The central Glycine (GLY) residue is displayed in light‐green/cyan stick representation. Due to its small side chain, GLY fits seamlessly into the existing local packing space. The surrounding network of interatomic interactions (indicated by dashed lines) is widely distributed and forms stable noncovalent contacts with neighboring residues such as ASN, LEU, and LYS, maintaining the structural integrity of the local environment. (b) MT RPGR (GLY to VAL): Upon substitution of GLY with the bulky Valine (VAL, highlighted in green sticks), the local steric landscape becomes highly constrained. In contrast to the WT, a dense cluster of red dashed lines emerges, explicitly indicating severe steric clashes and unfavorable van der Waals overlaps between the newly introduced VAL side‐chain methyl groups and the surrounding backbone/side‐chain residues. The bulky nature of VAL forces a physical rearrangement of adjacent residues, disrupting the original stable interaction network. Biophysical interpretation: The visual evidence of steric clashes is substantiated by the calculated vibrational entropy change (*ΔΔ*SVib) derived from ENCoM normal mode analysis, which is −1.010 kcal/mol^−1^·K^−1^. This significantly negative *ΔΔ*S value indicates that the mutation drastically restricts the conformational flexibility and vibrational dynamics of the local protein matrix. Consequently, the loss of local dynamic freedom combined with the introduced steric hindrance explains the predicted thermodynamic destabilization of the mutant RPGR protein compared to the wild‐type.

**Figure 4:**
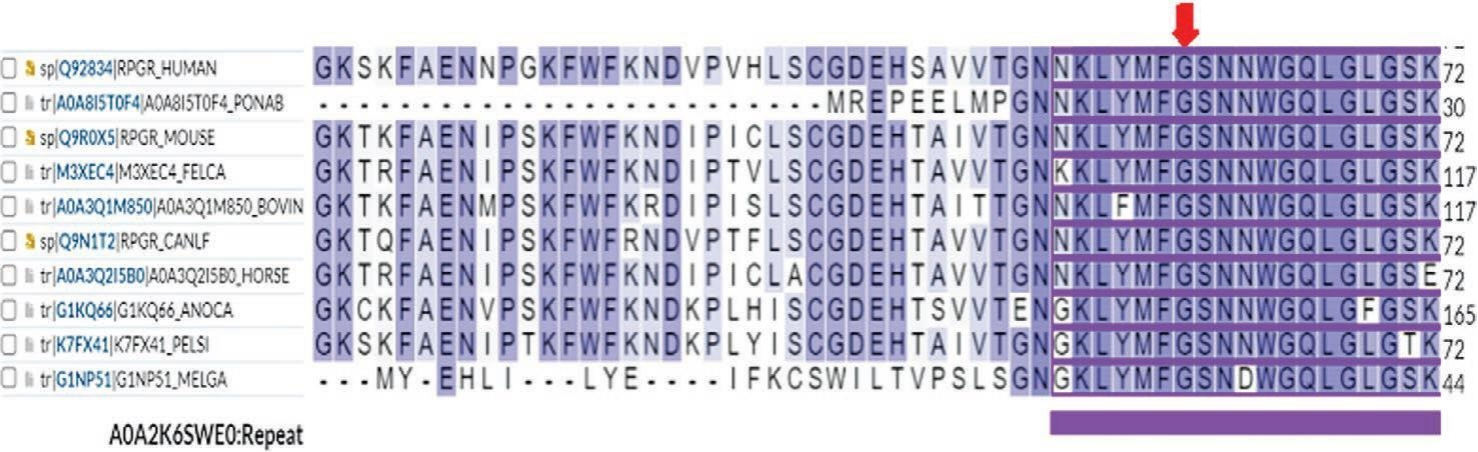
Conservation of the interested domain among different species. This data shows that the region is highly conserved among different species. Mutated residue is shown by red arrow (p.G65V).

Comparative structural analysis revealed a *ΔΔ*G value of −0.498 kcal/mol and a *ΔΔ*G SDM value of −1.440 kcal/mol, indicating decreased protein stability. Additionally, the *ΔΔ*SVib ENCoM value was −1.010 kcal/mol·K^−1^, suggesting diminished molecular flexibility, which may disrupt protein functionality (Figure 3). The RCC1 domain, which contains the identified variation, is highly conserved across species, as shown by bioinformatic analysis (Figure 4). This conservation implies that any alteration in this region would significantly impact the functioning of the encoded protein.

Prior research has elucidated mutations in the *RPGR* gene, highlighting the essential interaction between *RPGR* and *RPGRIP1*. This indicates that disruptions in RPGR functionality may impede this interaction, thereby playing a significant role in the pathogenesis of retinal degeneration associated with X‐linked RP.

This de novo variant was examined in a cohort of 430 healthy individuals from the Iranian–Azeri Turkish ethnic group, where it was not detected, resulting in a frequency of zero in our cohort. Furthermore, despite the region being covered in public population databases, including gnomAD and the 1000 Genomes Project, this variant has not been reported in any of these datasets.

Collectively, these findings provide substantial evidence supporting the potential pathogenicity of the variant. The absence of the mutation in both our control group (PS4), the occurrence of the variant in a de novo state (PP1), and the patient′s phenotype, which is highly specific for a disease with RPGR gene etiology (PP4), along with its predicted functional consequences, strongly suggest the pathogenicity of the identified de novo mutation (NC_000023.11) (NM_001034853.2) c.194G>T (p.G65V).

## 5. Discussion

The identification of mutations in the *RPGR* gene has garnered increased attention due to its association with RP. Previous studies have documented a variety of mutations within the RPGR gene, emphasizing the critical role of the RCC1 domain and the interaction between RPGR and RPGRIP1. The *RPGR* gene encodes a protein featuring a series of six RCC1‐like domains (RLDs), which are characteristic of the highly conserved guanine nucleotide exchange factor family of proteins. These factors play a crucial role in regulating the activity of small G‐proteins by facilitating the exchange of GDP for GTP, thereby activating the G‐protein signaling cascade [24].

In this study, we report a de novo mutation in the *RPGR* gene (c.194G>T) identified in a male patient with RP. Mutations in the *RPGR* gene are strongly associated with X‐linked RP, a severe form of inherited retinal degeneration. The disruption of RPGR protein function, particularly its effects on RPGRIP1 and photoreceptor viability, is believed to be a key pathogenic mechanism underlying the retinal degeneration observed in X‐linked RP patients. The RPGRIP1 protein is essential for the proper localization of RPGR to the connecting cilium of photoreceptors, a critical structure linking the inner and outer segments of these cells. Thus, any disruption of the RPGR protein could significantly impact its interaction with RPGRIP1, potentially resulting in a loss of essential RPGR functions.

To contextualize our molecular findings within the existing clinical literature, we compared our results with previously reported cases of the p.Gly65Asp (c.194G>T) mutation (PMIDs: 23372056, 21857984, and 20861475). Fahim et al. [25] (PMID: 21857984) demonstrated that RPGR mutations located in exons 1–14, which include the p.Gly65Asp residue, are typically associated with a more severe retinal phenotype compared with those in the ORF15 region, despite notable inter‐ and intrafamilial clinical variability. Although detailed longitudinal clinical metrics were not available in our study, the early‐onset and severe presentation in our male proband is consistent with this documented genotype–phenotype correlation for exon 1–14 mutations. Furthermore, Churchill et al. [26] (PMID: 23372056) highlighted that a substantial proportion (8.5%) of families initially classified as autosomal dominant RP (adRP) actually harbor X‐linked RPGR or RP2 mutations. Importantly, our identification of p.Gly65Asp as a de novo event in a male patient represents a distinct pathomechanism from the familial adRP misclassification described by Churchill et al.; nevertheless, it reinforces the critical message that RPGR mutations must be considered in all male RP cases, regardless of family history. Finally, Bowne et al. [27] (PMID: 20861475) emphasized the utility of next‐generation sequencing (NGS) in uncovering causative mutations in adRP; similarly, our NGS‐based identification, combined with extensive in silico functional predictions (mRNA stability and protein dynamics), extends their methodological approach by providing a comprehensive structural and functional rationale for the pathogenicity of this specific variant. Although these three reports provide valuable clinical and genetic context, the present study is distinctive in reporting p.Gly65Asp as a de novo mutation rather than a familial inherited one.

Through extensive bioinformatics analysis and cross‐species sequence comparisons, we confirmed that the glycine residue at the site of the identified genetic variant is highly conserved across various species, including humans, mice, chimpanzees, dogs, horses, cows, and cats. The evolutionary conservation of this specific amino acid strongly suggests its importance in the protein′s functional role. Alterations to conserved amino acids are often detrimental, as they can disrupt critical structural elements or interfere with essential biochemical properties of the protein. Therefore, the change in the glycine residue at the site of the identified variant is likely to lead to significant modifications in the structure and/or function of the affected protein, which is further supported by the findings presented in Figure 4.

Although our mRNA bioinformatics analysis indicated that the *p* value exceeded 0.05, significant changes were observed in the minimum free energy of the mutant and wild‐type mRNA structures. Specifically, the minimum free energy of the wild‐type mRNA transcript was −91.40 kcal/mol, whereas the variant resulted in a minimum free energy of −91.20 kcal/mol. This shift suggests alterations in the overall thermodynamic stability and folding dynamics of the mRNA molecule, which could have profound implications for its function.

To conduct a more comprehensive examination of the potential impacts of the identified genetic variant, we performed a detailed analysis of the transcribed mRNA structure and stability. Further, in silico analysis with the HSF web‐based tool indicated that the genetic variant likely activates a cryptic donor site within exon 3, increasing splice site strength by approximately 69%. However, empirical in vitro examinations, such as RT‐PCR or minigene assays, would be necessary to definitively ascertain whether this new donor site disrupts the normal mRNA splicing process.

Due to the limitations in our study regarding functional and objective testing, we could not conduct in vivo tests to precisely assess the impact of mutations in the gene. Further investigations in this area would be valuable.

To explore the potential structural and functional consequences of the identified genetic variant, we conducted comprehensive bioinformatics analyses using specialized tools. The DynaMut web application enabled us to perform a comparative analysis of the predicted structural and dynamic properties of the wild‐type and mutant protein forms. The DynaMut analysis revealed that the amino acid substitution resulting from the genetic variant induces significant changes to the dynamic behavior and overall stability of the protein structure. Specifically, we observed a *ΔΔ*G value of −0.498 kcal/mol when comparing the mutant and wild‐type proteins, with a *ΔΔ*G score calculated using the SDM algorithm of −1.440 kcal/mol.

These negative *ΔΔ*G values indicate that the genetic variant destabilizes the protein structure, rendering it less thermodynamically stable compared to the wild‐type form. Furthermore, the DynaMut analysis indicated a *ΔΔ*SVib of −1.010 kcal/mol·K^−1^, suggesting that the mutation decreases the overall flexibility and dynamic mobility of the protein molecule. The observed decreases in protein stability and flexibility, illustrated in Figure 3, are likely to have significant functional consequences.

ClinVar contains a single entry for this specific RPGR variant (accession: VCV003724221.2), reporting an association with RP, which further validates our findings. The identified RPGR gene variant (NC_000023.11)(NM_001034853.2): c.194G>T has been previously reported as pathogenic in the ClinVar database. A key advantage of the present study is that we demonstrate this variant to be a de novo mutation in our patient. This de novo occurrence provides strong supportive evidence for its pathogenicity (PS2/PM6) and suggests a specific mechanism of disease onset in this case that warrants further investigation.

## Funding

No funding was received for this manuscript.

## Conflicts of Interest

The authors declare no conflicts of interest.

## Data Availability

The data that support the findings of this study are available from the corresponding author upon reasonable request.
